# Percutaneous Microwave Ablation of Hepatocellular Carcinoma As Bridge-to-Transplant Therapy in a High-Risk Patient With Alpha-1 Antitrypsin Deficiency and Factor V Leiden Mutation: A Case Report

**DOI:** 10.7759/cureus.104574

**Published:** 2026-03-02

**Authors:** Zuhayr Khan, Mahsum Jafri, Constantino G Lambroussis, Zohha Khan, Mehreen Khan

**Affiliations:** 1 General Medicine, Lake Erie College of Osteopathic Medicine, Elmira, USA; 2 Internal Medicine, Lake Erie College of Osteopathic Medicine, Elmira, USA; 3 Osteopathic Medicine/Family Medicine, Lake Erie College of Osteopathic Medicine, Elmira, USA; 4 Public Health, Saint Mary's University of Minnesota, Minneapolis, USA

**Keywords:** alpha-1 antitrypsin, child-pugh class, factor v leiden deficiency, hepatocellular carcinoma (hcc), hypercoagulable state, liver cirrhosis (lc)

## Abstract

The use of percutaneous microwave ablation has been established as a treatment for early-stage hepatocellular carcinoma (HCC). HCC can often arise in the setting of cirrhosis and often requires locoregional therapy to ablate the area as a bridge for the ultimate treatment of liver transplantation. The challenge is its use in patients with decompensated cirrhosis and hypercoagulable states, as these high-risk populations can lead to other major complications, requiring anticoagulation, which remains challenging. In this case report, a 60-year-old woman with a history of alpha-1 antitrypsin deficiency-related cirrhosis presented with Child-Pugh class B (score 9) liver disease, thrombocytopenia, portal hypertension with esophageal varices, and factor V Leiden mutation. She underwent successful image-guided microwave ablation of a segment 7/8 Liver Imaging Reporting and Data System (LI-RADS) 5 HCC lesion as a bridge to liver transplantation, performed by interventional radiology. In this ablation, she had significant procedural risk factors, but with multidisciplinary planning, there was only a minor non-distressing hematoma noted, and overall allowed effective tumor treatment without major complications. This case highlights the practicality of microwave ablation in high-risk patients and emphasizes the critical role of interventional radiology in expanding therapeutic options for transplant candidates with limited alternatives.

## Introduction

Hepatocellular carcinoma (HCC) is the most common primary malignancy of the liver and often develops in patients with chronic liver disease and cirrhosis, as in our patient [1]. The ultimate treatment with liver transplantation offers the best long-term choice for survival (as long as they’re eligible) in patients as it addresses both for the potential of tumor burden and the associated liver dysfunction; on the other hand, the public health issue of prolonged wait times for a liver transplant places patients at risk for tumor progression beyond transplant criteria, thus necessitating ablation to give more time [2]. The use of locoregional therapies such as microwave thermal ablation is therefore frequently employed as the choice of ablation type as a bridge to transplantation to maintain tumor control with its high success rates [3].

Microwave ablation (MWA) has emerged as a preferred ablative modality due to its ability to generate higher intratumoral temperatures, larger ablation zones, and reduced susceptibility to heat-sink effects compared with radiofrequency ablation [4]. Current clinical practice guidelines support ablation for tumors ≤3 cm in patients with preserved liver function [5]. This is common practice even though evidence is limited regarding its safety in patients with decompensated cirrhosis and other risk factors (such as portal hypertension, thrombocytopenia, and concurrent anticoagulation-dabigatran use) [6,7].

Patients with inherited hypercoagulable disorders such as factor V Leiden mutation can pose additional complications due to the competing risks of thrombosis and bleeding, particularly in this setting of cirrhosis-related coagulopathy [8]. Here, we present a case demonstrating the successful use of percutaneous MWA as a bridge-to-transplant strategy in a patient with multiple high-risk conditions that interplay with each other, which emphasizes interventional radiology’s role in decision-making and technical considerations.

This case highlights the technical and peri-procedural considerations of performing MWA in a transplant candidate with Child-Pugh class B (score 9) cirrhosis, Albumin-Bilirubin (ALBI) grade 3 liver dysfunction, and concurrent inherited thrombophilia requiring anticoagulation.

## Case presentation

A 60-year-old female was referred to interventional radiology for management of HCC in the setting of her complex presentation of cirrhosis secondary to alpha-1 antitrypsin deficiency. Her medical history was lengthy and significant for decompensating cirrhosis complicated by portal hypertension. Moreover, this also led to esophageal varices with prior hemorrhage status post recent band ligation. She also has a history of factor V Leiden mutation, which has led to prior deep vein thrombosis and is maintained on dabigatran therapy for prevention/management. She has additional comorbidities, including hypothyroidism, obesity, obstructive sleep apnea, and recurrent non-melanoma skin cancers.

**Figure 1:**
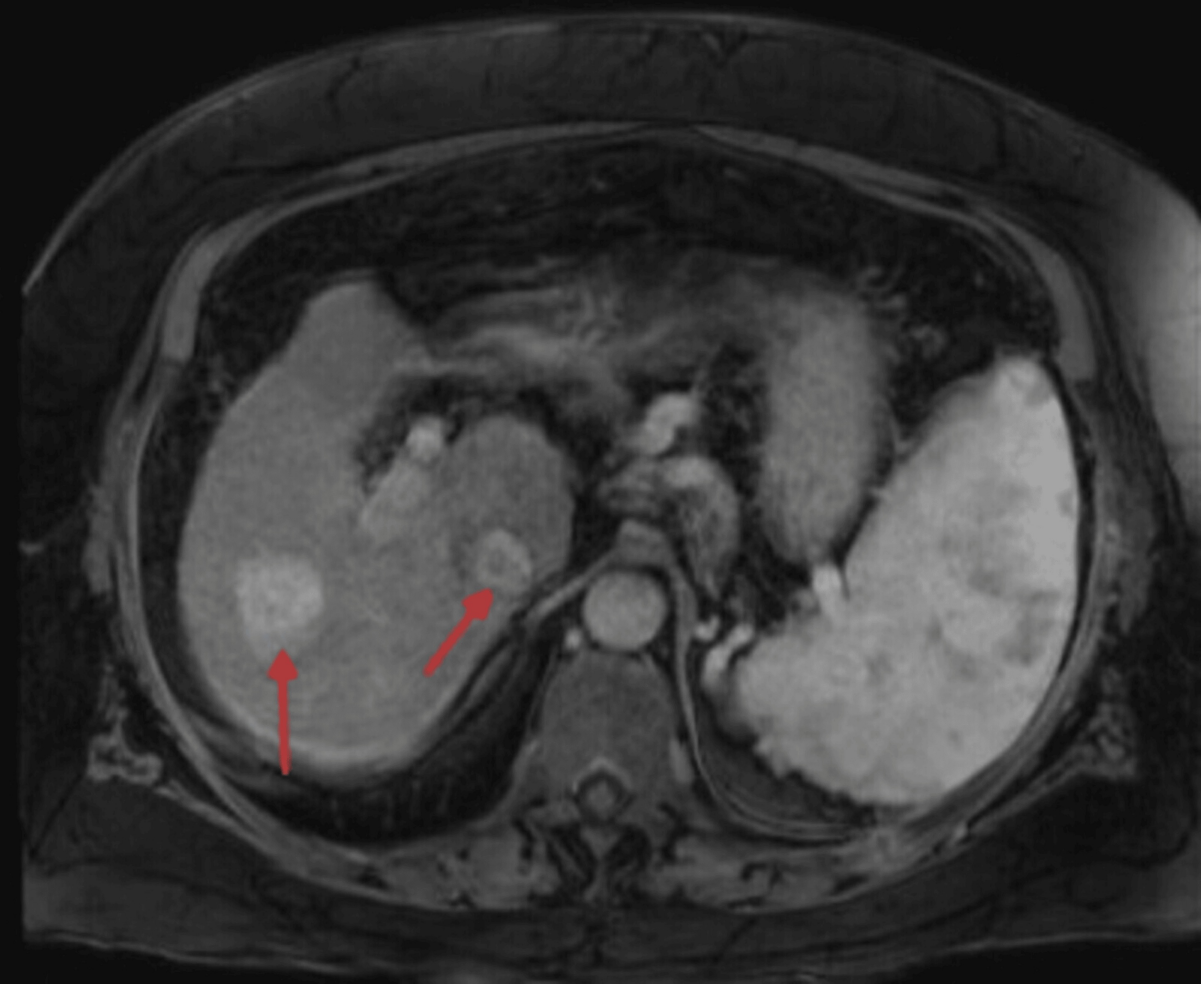
Preprocedural magnetic resonance imaging (MRI) of the liver demonstrating hepatocellular carcinoma. Axial contrast-enhanced T1-weighted imaging shows a 3.4-cm arterially enhancing lesion at the junction of hepatic segments 7 and 8, with intrinsic T1 hyperintensity and imaging features consistent with a Liver Imaging Reporting and Data System (LI-RADS) 5 hepatocellular carcinoma (red arrows). Interval growth on serial imaging prompted consideration of locoregional therapy as a bridge to liver transplantation.

The surveillance imaging of the liver, as seen in Figure 1, demonstrated a progressively enlarging lesion at the junction of hepatic segments 7 and 8. In Figure 1, a magnetic resonance imaging revealed a 3.4-cm arterially enhancing lesion with intrinsic T1 hyperintensity and features consistent with an LR-5 observation. The lesion had increased in size from 2.7 cm over a one-month interval, accompanied by rising alpha-fetoprotein levels from 28.3 ng/mL to 49.9 ng/mL (normal reference range of 0-8 ng/mL). Her laboratory evaluation was also consistent with Child-Pugh class B9 and ALBI grade 3 (see Table 1).

**Table 1:** Laboratory values supporting Child-Pugh Class B9 and ALBI Grade 3 ALBI: Albumin-Bilirubin; INR: international normalized ratio; AST: aspartate aminotransferase; ALT: alanine aminotransferase

Laboratory Parameter	Patient Value	Reference Range
Total bilirubin (mg/dL)	3.8	0.2-1.2
Serum albumin (g/dL)	2.8	3.5-5.0
INR	1.54	0.8-1.2
Platelet count (×10^9^/L)	80	150-400
AST (U/L)	71	10-40
ALT (U/L)	48	7-56

Due to the patient's decompensated liver function and other complications, surgical resection was contraindicated. Other options for management were considered, such as transarterial chemoembolization and radioembolization, but were deferred due to concern for further hepatic decompensation. After a multidisciplinary discussion among many care teams, it was decided to go for a minimally invasive procedure of percutaneous MWA, as it was selected as the most suitable bridge-to-transplant option in this patient with a lower risk of complications.

**Figure 2:**
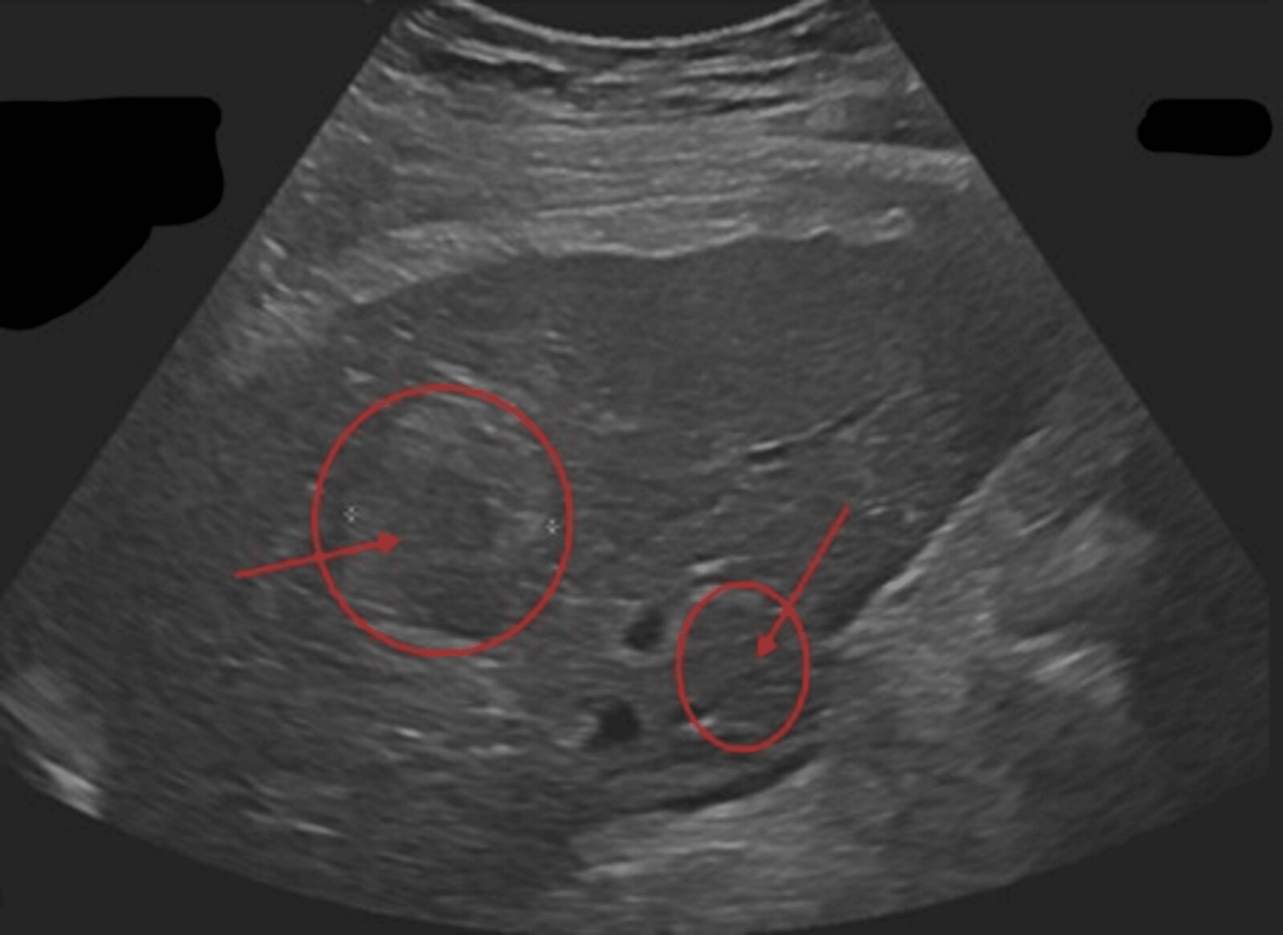
Ultrasound visualization of hepatocellular carcinoma prior to ablation. Grayscale ultrasound imaging demonstrates a well-visualized heterogeneous lesion in the posterior right hepatic lobe (segments 7 and 8), allowing real-time sonographic guidance for percutaneous probe placement (red annotations). Adequate lesion visualization was essential to ensure safe and accurate microwave ablation in this high-risk patient.

**Figure 3:**
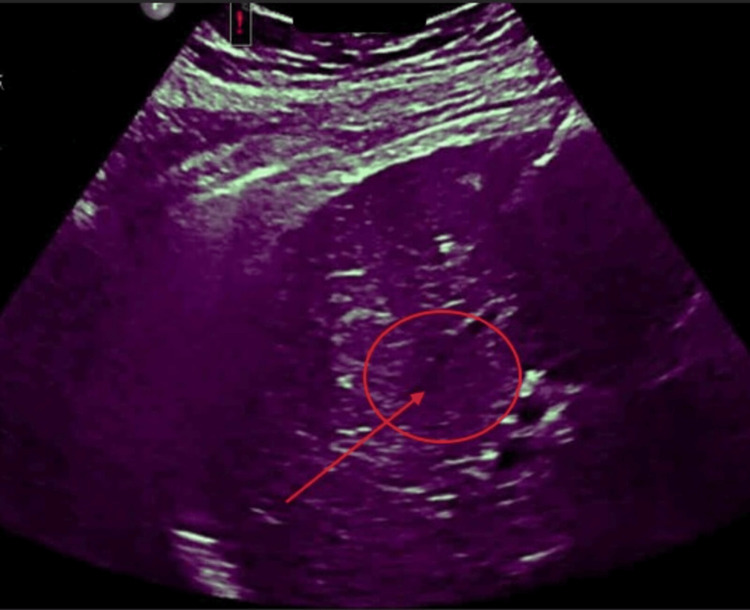
Post-ablation color Doppler ultrasound demonstrating the ablation zone. Color Doppler imaging performed immediately following microwave ablation shows the expected non-enhancing ablation zone encompassing the treated lesion in hepatic segments 7 and 8 (red annotations). A small perihepatic hematoma was noted, which remained stable on serial imaging and did not require intervention.

Prior to the procedure, the patient had been taking dabigatran for four doses to prevent any excess bleeding. On procedure day, the patient underwent general anesthesia, and an ultrasound was used to visualize the lesion and guide probe placement, as seen in Figure 2. To conduct the ablation, two MWA applicators were placed in inferomedial and superolateral positions within the tumor to ensure adequate coverage. The ablation was performed using 120 W for eight minutes, followed by escalation to 160 W for an additional four minutes. Tract cauterization was performed upon applicator removal. Post-ablation color doppler ultrasound (as seen in Figure 3) demonstrated an adequate ablation zone with a small perihepatic hematoma, which remained stable following manual compression and ultrasound surveillance. The patient was admitted overnight for monitoring, where she experienced no significant complications and was allowed to be discharged and resumed anticoagulation 24 hours later after stable hemoglobin trends (no bridging anticoagulation was administered).

## Discussion

This case illustrates the feasibility and practicality of MWA as a bridge-to-transplant therapy in a patient with significant procedural risk factors. While ablation therapy is well established in patients with preserved liver function, its role in Child-Pugh B and C patients remains controversial due to concerns for post-procedural liver failure and bleeding [6,7].

When comparing thermal ablation with transarterial therapies, it’s noted that thermal ablation offers the advantage of limiting non-target hepatic injury, an important consideration in patients with marginal liver reserve [9]. It was also demonstrated that transarterial chemoembolization can exacerbate hepatic dysfunction in patients with advanced cirrhosis [9]. The author also noted that other therapies that are radiation-based can carry an increased risk of radiation-induced liver disease [9]. Given this context, MWA may represent the least hepatotoxic locoregional option [9].

MWA also provides technical advantages over radiofrequency ablation due to its faster heating and more predictable ablation zones properties, which are particularly relevant for the lesions seen in our patient, as it’s in a challenging location, such as hepatic segment 7/8, as seen in Figure 1 [10]. It has also been emphasized that ablating lesions adjacent to the diaphragm can be technically challenging due to potential respiratory complications, thereby reconfirming the importance of careful probe placement and power modulation. Lesions in the posterior superior liver segments (7 and 8) are technically challenging due to limited sonographic windows, respiratory motion, and proximity to the diaphragm, increasing the risk of incomplete ablation or non-target injury [10]. Additionally, a systematic review demonstrated comparable or superior local tumor control with MWA compared with radiofrequency ablation [11].

The management of anticoagulation in patients with the factor V Leiden mutation presents additional complexity. In a study, they mention that although cirrhosis is associated with a “rebalanced” hemostatic state, bleeding and thrombotic risks coexist [12]. Thus, facilitating temporary interruption of dabigatran when combined with tract cauterization and close post-procedural monitoring allowed safe ablation without a clinically significant hematoma in this patient.

## Conclusions

In this case report, we presented how percutaneous MWA can be safely and effectively performed as a bridge-to-transplant therapy, especially in carefully selected high-risk patients with HCC. Even in the setting of decompensated cirrhosis and inherited hypercoagulable states requiring anticoagulation, thoughtful multidisciplinary planning and technical optimization can help mitigate procedural risks. This case also underlines the expanding role of interventional radiology in providing oncologic therapies to transplant candidates with limited treatment options due to the minimally invasive nature of the field. Also, as a single case with limited follow-up, these findings should be interpreted cautiously and are not intended to imply generalizable safety across all high-risk populations.
